# Outcomes with luspatercept in patients with β-thalassemia: a systematic review and meta-analysis

**DOI:** 10.1007/s00277-026-07151-5

**Published:** 2026-06-25

**Authors:** Nihal Babiker, Eslam Abady, Hafsa Shahid, Wania Ur Rehman, Faris Babker, Menna Sarhan, Hind El Azzazi, Mohamedelborae Abdalgafar Elimam Mohamed, Hafiz Muhammad Hannan Javed, Yahya Samadi

**Affiliations:** 1 Faculty of Medicine, Zagazig University, Zagazig, Egypt; 2 Faculty of Medicine, Tanta University, Tanta, Egypt; 3 Tanta University Hospitals, Tanta, Egypt; 4 The MetroHealth System, Cleveland, OH United States; 5 King Edward Medical University, Lahore, Punjab Pakistan; 6 Zagazig University, Zagazig, Egypt; 7 Mohammed V University, Rabat, Morocco; 8 TidalHealth Peninsula Regional Medical Center, Salisbury, MD United States; 9 Department of Curative Medicine, Faculty of Medicine, Kateb University, Kabul, Afghanistan

**Keywords:** β-thalassemia, Luspatercept, Transfusion independence, Mmeta-analysis, Rythropoiesis

## Abstract

β-thalassaemia is a genetic blood disorder characterized by ineffective erythropoiesis and chronic anaemia, often requiring lifelong red blood cell (RBC) transfusions. Luspatercept, an erythroid maturation agent, has emerged as a potential therapy to reduce transfusion burden in affected adults. To assess the clinical efficacy and safety of luspatercept in managing adult patients with β-thalassemia. We conducted a systematic review and meta-analysis of randomized controlled trials (RCTs) evaluating luspatercept in adult patients with β-thalassemia. A comprehensive search of PubMed, Scopus, Embase, Cochrane Library, and ClinicalTrials.gov was performed for studies published up to May 2025. Only English-language RCTs were included. Data were extracted on transfusion burden, hemoglobin response, and adverse events. Risk of bias was assessed using the Cochrane ROB 2 tool. Pooled estimates were calculated using a random-effects model with the Freeman–Tukey double arcsine transformation. Heterogeneity was assessed using the I² statistic. Out of 587 identified records, three RCTs and one single-arm trial (*n* = 608 patients) met the inclusion criteria. Of these, 447 patients received luspatercept and 161 received placebo. Luspatercept was associated with a ≥ 33% reduction in RBC transfusion burden in 58% (95% CI: 0.33–0.79) and a ≥ 50% reduction in 40% (95% CI: 0.25–0.58) of patients. Transfusion independence was achieved in 36% (95% CI: 0.13–0.69). Common adverse events included headache (32%), bone pain (33%), arthralgia (22%), and back pain (20%). Grade ≥ 3 adverse events occurred in 21% (95% CI: 0.09–0.41). Overall, luspatercept effectively reduced transfusion needs with an acceptable safety profile. Luspatercept significantly reduces transfusion burden in patients with β-thalassemia and demonstrates a manageable safety profile. These findings support luspatercept as an effective and well-tolerated therapeutic option that meaningfully reduces transfusion burden, potentially decreasing iron overload and improving quality of life for patients with β-thalassemia.

**Supplementary Information:**

The online version contains supplementary material available at https://doi.org/10.1007/s00277-026-07151-5.

## Introduction

Thalassemias are among the most common inherited autosomal recessive disorders worldwide. The global burden of thalassemia is substantial, with an estimated 1.31 per 100,000 population affected, and over 100,000 infants born annually with severe hemoglobinopathies, predominantly thalassemia and sickle cell disease [1]. The World Health Organization recognizes thalassemia as a major health burden in many regions [2]. Beta-thalassemia, the most common type, is characterized by a deficiency or total lack of beta-globin chains, leading to an excess of free alpha chains that precipitate in red blood cells, causing hemolysis, chronic anemia, and iron overload [3, 4].

Beta-thalassemia manifests along a spectrum of severity. Beta-thalassemia minor is usually asymptomatic, whereas beta-thalassemia intermedia commonly presents with anemia, fatigue, shortness of breath, dark urine, and bone deformities. Beta-thalassemia major, the most severe form, is typically transfusion-dependent and leads to serious complications such as liver damage and congestive heart failure [5]. Iron chelation and blood transfusion remain the mainstay of therapy, but these approaches are burdensome and associated with complications including heart failure, diabetes, infections, and thrombosis [6], highlighting the need for safer treatment options such as luspatercept.

Luspatercept, FDA-approved in 2019, is a lab-designed protein that enhances red blood cell production by inhibiting SMAD2/3 signaling, a pathway that is overactive in beta-thalassemia and disrupts late-stage erythropoiesis [7, 8]. Clinical studies have shown that luspatercept significantly reduces transfusion burden and increases hemoglobin concentration [8, 9]. The safety profile includes mild and manageable side effects such as headache, bone pain, arthralgia, dizziness, hypertension, and hyperuricemia [8]. This meta-analysis updates previous evidence with a larger number of studies and focuses on both efficacy and comprehensive safety outcomes.

## Methods

### Study design

**Fig. 1:**
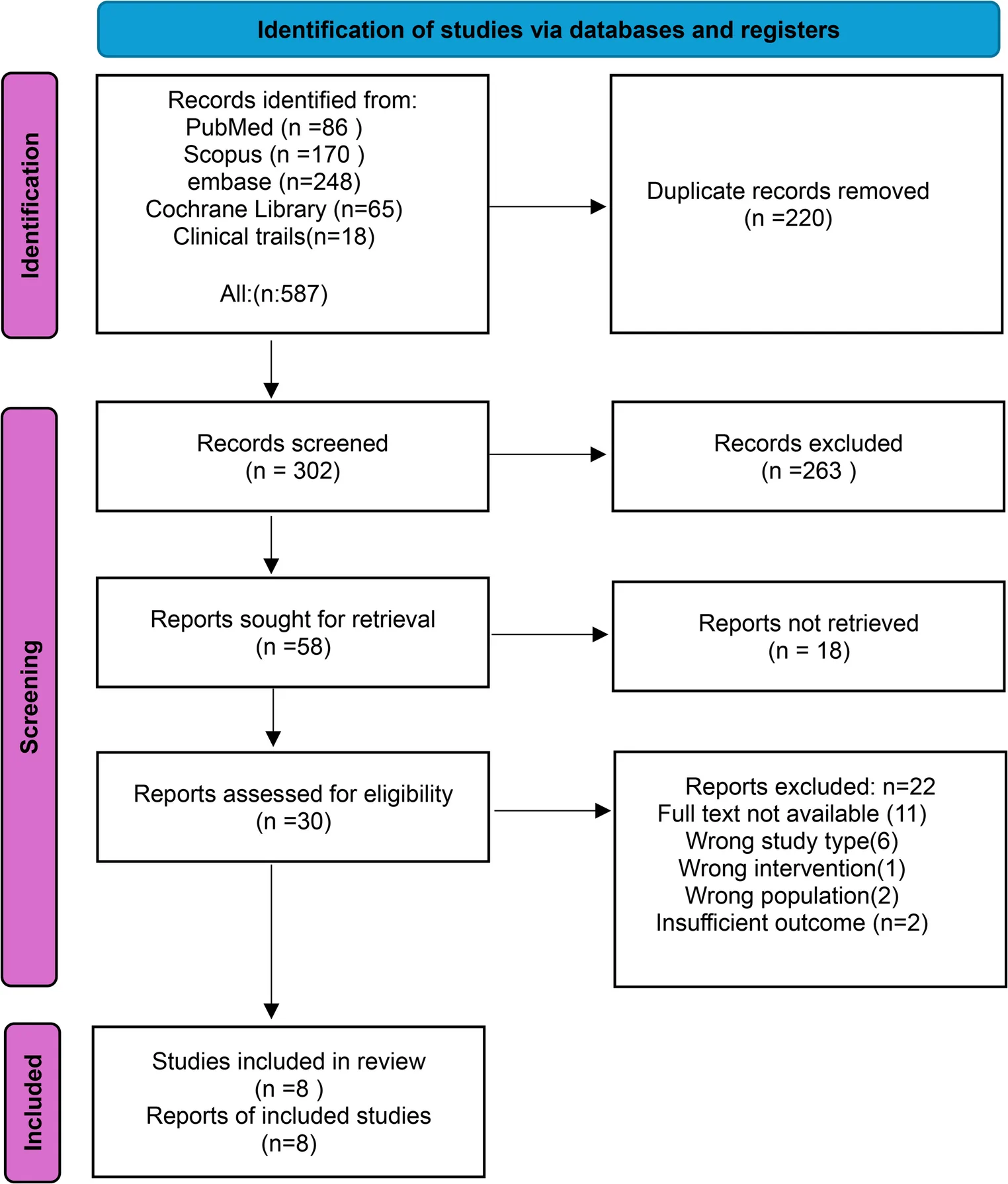
PRISMA 2020 flow diagram of study selection process. From 587 records identified across PubMed (*n* = 86), Scopus (*n* = 170), Embase (*n* = 248), Cochrane Library (*n* = 65), and ClinicalTrials.gov (*n* = 18), 220 duplicates were removed. After screening 302 records, 263 were excluded. Fifty-eight reports were sought for retrieval, of which 18 were not retrieved. Thirty reports were assessed for eligibility, and 22 were excluded (full text not available [11], wrong study type [6], wrong intervention [1], wrong population [2], insufficient outcome [2]). three RCTs and one single arm trial were included in the quantitative synthesis. Four additional studies were included in qualitative synthesis

This systematic review and meta-analysis followed the Preferred Reporting Items for Systematic Reviews and Meta-Analyses (PRISMA) guidelines (Fig. 1). The study protocol was registered at PROSPERO (CRD420251053194).

### Eligibility criteria

Inclusion criteria were: (1) studies evaluating luspatercept in patients with beta thalassemia (transfusion-dependent or non-transfusion-dependent) compared to placebo or standard care; (2) RCTs, cohort studies, or clinical trials (prospective or retrospective); (3) studies published in English. No time restrictions were applied. Exclusion criteria were: (1) studies on other hemoglobinopathies without beta-thalassemia-specific data; (2) preclinical studies; (3) case reports or small case series (< 10 patients); (4) reviews, editorials, commentaries, or abstracts without full data; (5) non-English publications without translation; (6) duplicate publications.

### Information sources and search strategy

A comprehensive literature search was conducted across PubMed, Cochrane, Embase, Scopus, and ClinicalTrials.gov to retrieve all relevant studies published up to May 2025, using the following search strategy: (“luspatercept” OR “luspatercept-aamt” OR “RAP-536” OR “ACE-536” OR “reblozyl” OR “TGF-beta superfamily ligand trap” OR “transforming growth factor-beta superfamily ligand trap” OR “erythroid maturation agent”) AND (“thalassemia” OR “thalassaemia” OR “β-thalassemia” OR “beta thalassemia” OR “cooley’s anemia” OR “mediterranean anemia” OR “β-thalassemia major” OR “β-thalassemia minor” OR “β-thalassemia intermedia” OR “β-thalassemia trait” OR “beta-thalassemia major” OR “beta-thalassemia minor” OR “beta-thalassemia intermedia” OR “transfusion-dependent thalassemia” OR “TDT” OR “non-transfusion-dependent thalassemia” OR “NTDT” OR “hydrops fetalis” OR “hemoglobin H disease” OR “HbH disease”). The detailed search strategy with results per database is provided in Supplementary Table 1.

### Study selection and data extraction

References were downloaded to reference management software, and duplicates were removed. The remaining references were imported into Rayyan for abstract screening. Three investigators independently screened titles and abstracts, then reviewed full texts for eligibility. Data extraction was performed independently by two reviewers using a standardized form, capturing study characteristics, patient characteristics, efficacy outcomes (≥ 33% and ≥ 50% reduction in RBC transfusion burden, transfusion independence, hemoglobin increase ≥ 1.0 g/dL), and safety outcomes (grade ≥ 3 adverse events, bone pain, headache, myalgia, arthralgia, musculoskeletal pain, back pain, injection site reactions, nausea, pyrexia, pain in extremity, asthenia). Discrepancies were resolved by consensus.

### Risk of bias assessment

Quality assessment was conducted independently by two investigators using the Cochrane ROB 2 tool for randomized controlled trials and ROBINS-I for non-randomized studies. Conflicts were resolved by discussion.

### Statistical analysis

All statistical analyses were performed using R software (version 4.2.1) with the meta and metafor packages. A random-effects model with the restricted maximum-likelihood (REML) estimator was used to calculate pooled proportions and 95% confidence intervals (CIs). The Freeman–Tukey double arcsine transformation was applied to stabilize variances. Heterogeneity was assessed using the I² statistic and Cochran’s Q test. For outcomes with substantial heterogeneity (I² > 50% or *p* < 0.10), study characteristics were examined. A leave-one-out sensitivity analysis was planned for outcomes with high heterogeneity. Publication bias was not formally assessed due to the small number of included studies (< 10).

## Results

### Study selection and baseline characteristics

The initial search identified 587 potential studies. After removing duplicates (*n* = 220), 302 records were screened, and 263 were excluded. Fifty-eight reports were sought for retrieval, of which 18 were not retrieved. Thirty reports were assessed for eligibility, and 22 were excluded (full text not available [11], wrong study type [6], wrong intervention [1], wrong population [2], insufficient outcome [2]). three RCTs and one single-arm trial met the inclusion criteria for quantitative synthesis, all published between 2019 and 2025. The total sample size was 608 patients, with 447 treated with luspatercept and 161 with placebo. Four additional studies (Panzieri 2024, Torti 2024, and two others) were included in qualitative synthesis but not in pooled analyses due to lack of control groups or outcome incompatibility. Figure 1 provides the PRISMA flow diagram, and Table 1 details the baseline characteristics.

**Table 1:** Baseline characteristics of included studies

Study	Year	Total patients (Luspatercept/Placebo)	Disease type	Follow-up duration
Piga 2019	2019	64 (32/32)	TDT	Not specified
Piga 2022	2022	64 (32/32)	TDT	Not specified
Cappellini 2022	2022	96 (64/32)	NTDT	Not specified
Cappellini 2025	2025	223 (149/74)	TDT	Not specified

*TDT*, transfusion-dependent thalassemia; *NTDT,* non-transfusion-dependent thalassemia

### Efficacy outcomes

#### ≥ 33% reduction in RBC transfusion burden

**Fig. 2:**
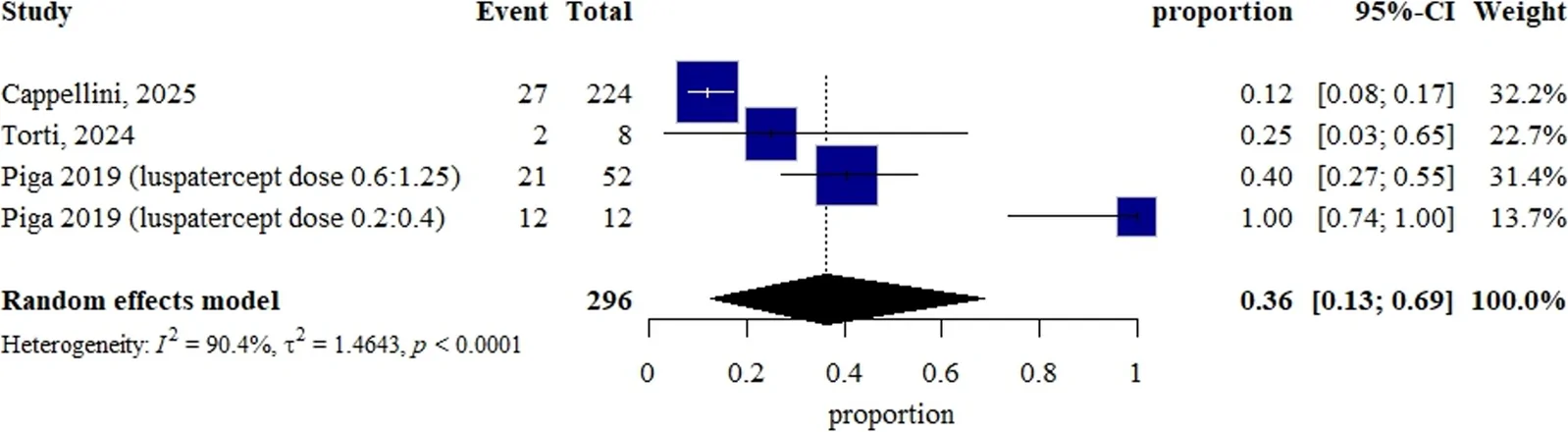
Forest plot of ≥33% reduction in RBC transfusion burden. Four studies (Piga 2019, Piga 2022, Cappellini 2025, Panzieri 2024). Pooled proportion: 0.58 (95% CI: 0.33–0.79). Heterogeneity: I² = 92.0%, *p* < 0.0001

All three RCTs and one single-arm trial reported this outcome. The pooled proportion was 0.58 (95% CI: 0.33–0.79), with high heterogeneity (I² = 92.0%, *p* < 0.0001) (Fig. 2).

#### ≥ 50% reduction in RBC transfusion burden

**Fig. 3:**
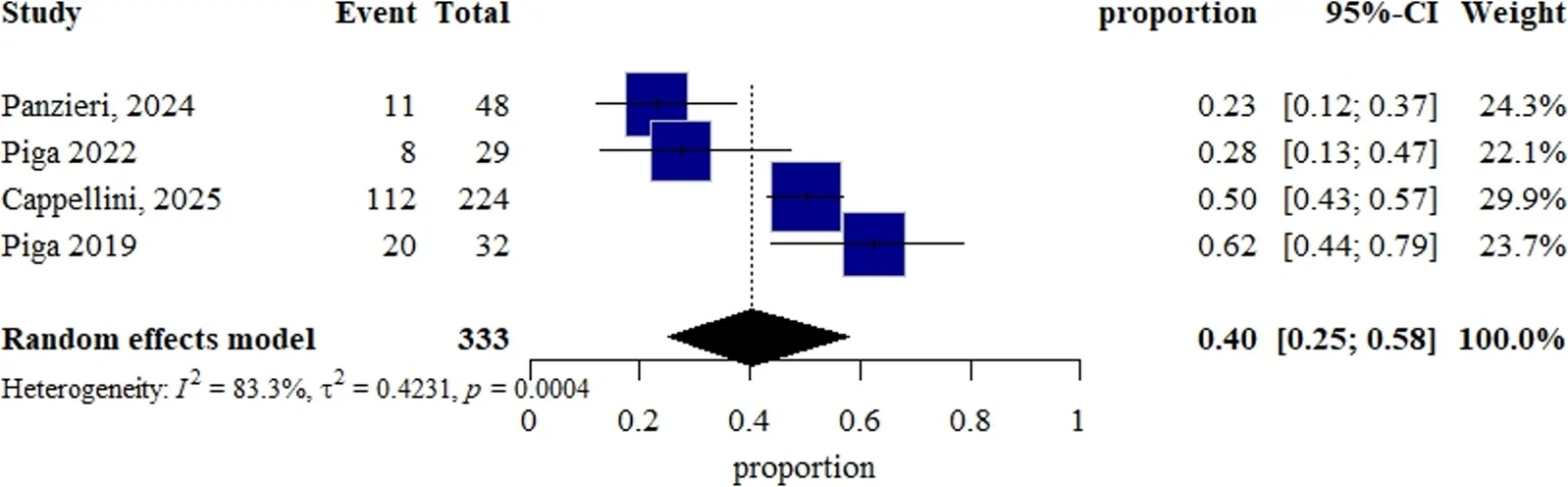
Forest plot of ≥50% reduction in RBC transfusion burden. Four studies (Piga 2019, Piga 2022, Cappellini 2025, Panzieri 2024). Pooled proportion: 0.40 (95% CI: 0.25–0.58). Heterogeneity: I² = 83.3%, *p* = 0.0004

All three RCTs and one single-arm trial reported this outcome. The pooled proportion was 0.40 (95% CI: 0.25–0.58), with high heterogeneity (I² = 83.3%, *p* = 0.0004) (Fig. 3).

#### Transfusion independence

**Fig. 4:**
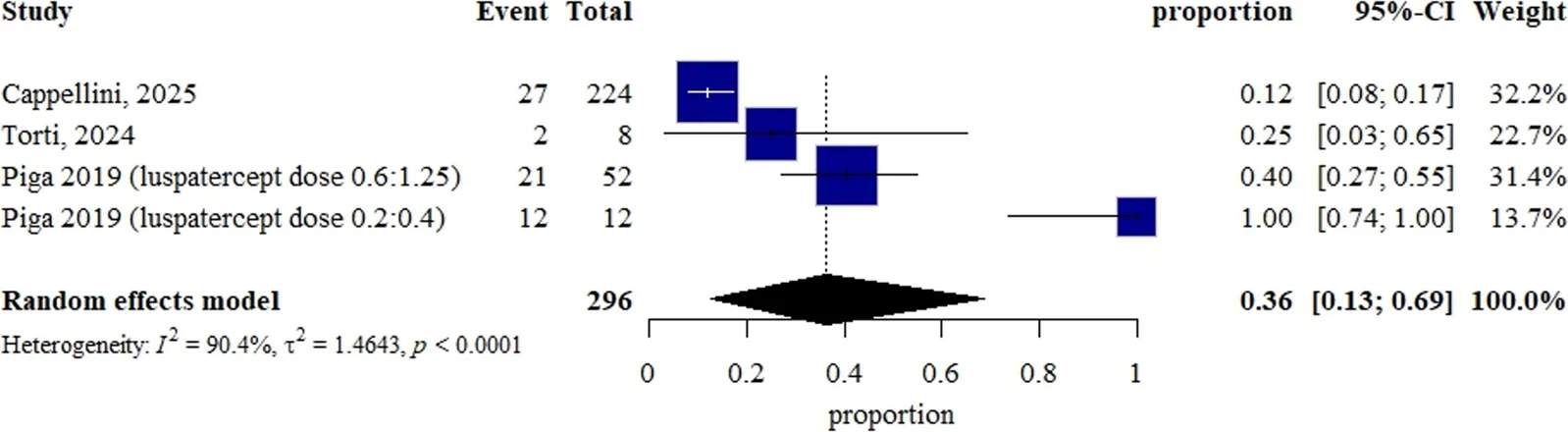
Forest plot of transfusion independence. Four studies (Piga 2019 [two dose groups], Cappellini 2025, Torti 2024). Pooled proportion: 0.36 (95% CI: 0.13–0.69). Heterogeneity: I² = 90.4%, *p* < 0.0001

Four studies (including dose groups from Piga 2019) reported this outcome. The pooled proportion was 0.36 (95% CI: 0.13–0.69), with high heterogeneity (I² = 90.4%, *p* < 0.0001) (Fig. 4).

#### Hemoglobin increase ≥ 1.0 g/dL

Three studies reported this outcome. The pooled proportion was 0.69 (95% CI: 0.53–0.81) (Supplementary Figure S11).

#### Clinical interpretation

The proportion of patients achieving a ≥ 33% reduction in transfusion burden (58%) and transfusion independence (36%) represents meaningful clinical benefit. For patients with lifelong transfusion dependency, these outcomes translate directly into reduced iron accumulation, lower chelation requirements, and potential improvements in quality of life.

### Safety outcomes

#### Headache 

**Fig. 5:**
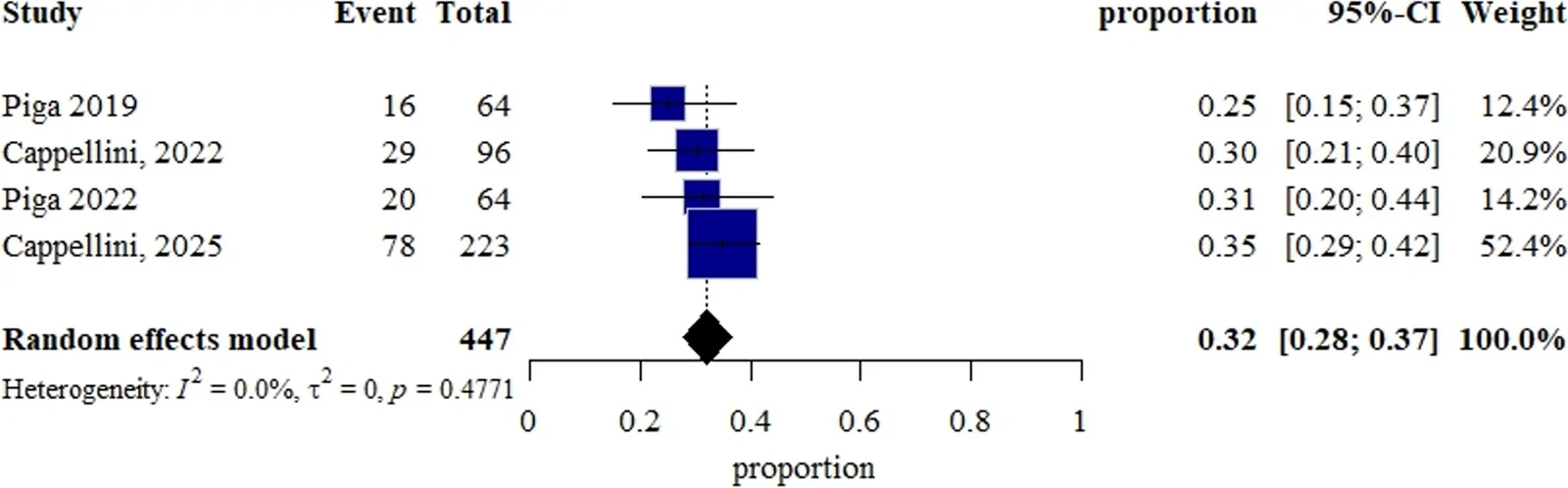
Forest plot of headache prevalence. Four studies (Piga 2019, Piga 2022, Cappellini 2022, Cappellini 2025). Pooled proportion: 0.32 (95% CI: 0.28–0.37). Heterogeneity: I² = 0.0%, *p* = 0.4771

All three RCTs and one single-arm trial reported headache. The pooled proportion was 0.32 (95% CI: 0.28–0.37), with no heterogeneity (I² = 0%, *p* = 0.4771) (Fig. 5).

#### Grade ≥ 3 adverse events

**Fig. 6:**
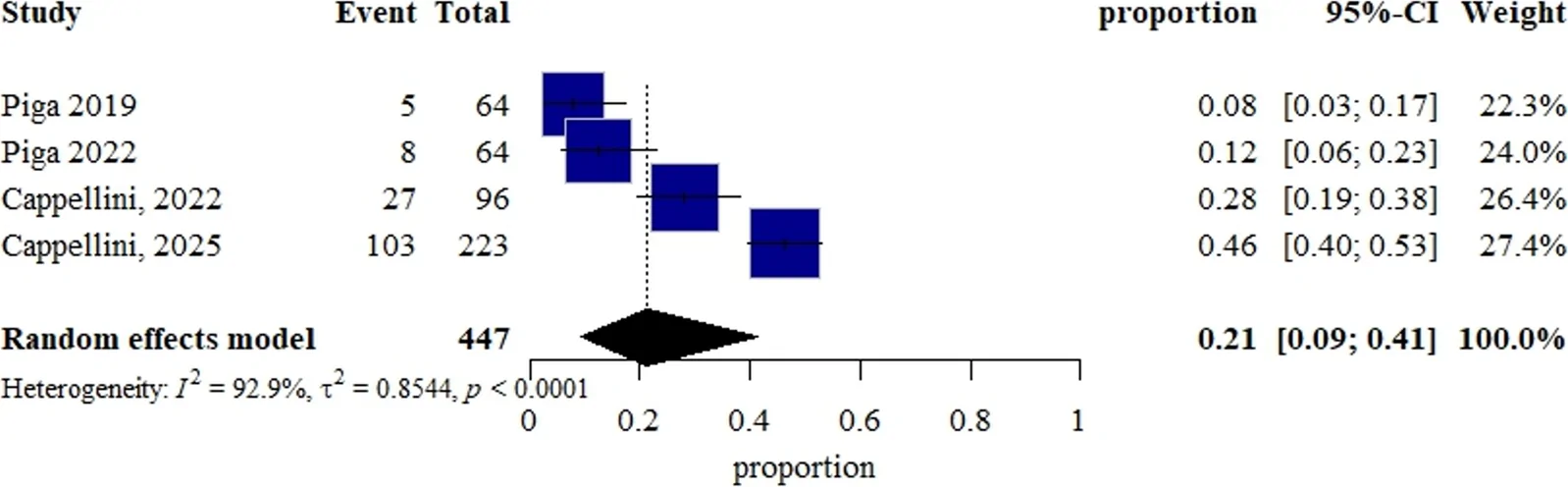
Forest plot of grade ≥3 adverse events. Four studies (Piga 2019, Piga 2022, Cappellini 2022, Cappellini 2025). Pooled proportion: 0.21 (95% CI: 0.09–0.41). Heterogeneity: I² = 92.9%, *p* < 0.0001

All four RCTs reported grade ≥ 3 AEs. The pooled proportion was 0.21 (95% CI: 0.09–0.41), with high heterogeneity (I² = 92.9%, *p* < 0.0001) (Fig. 6).

Other adverse events (all with pooled proportions and heterogeneity as shown in Table 2) included arthralgia (22%, I² = 9.9%), asthenia (12%, I² = 0%), back pain (20%, I² = 85.1%), bone pain (33%, I² = 76.1%), myalgia (16%, I² = 47.4%), musculoskeletal pain (11%, I² = 53.8%), injection site AEs (8%, I² = 56.4%), nausea (10%, I² = 54.2%), pain in extremity (13%, I² = 65.0%), and pyrexia (14%, I² = 76.7%). Forest plots for these outcomes are provided in Supplementary Figures S1–S10.

**Table 2:** Summary of efficacy and key safety outcomes

Outcome	Number of studies	Pooled proportion	95% CI	I² (%)	*P*-value
Efficacy					
≥ 33% reduction in RBC transfusion burden	4	0.58	0.33–0.79	92.0	< 0.0001
≥ 50% reduction in RBC transfusion burden	4	0.40	0.25–0.58	83.3	0.0004
Transfusion independence	4	0.36	0.13–0.69	90.4	< 0.0001
Hemoglobin increase ≥ 1.0 g/dL	3	0.69	0.53–0.81	—	—
Safety					
Headache	4	0.32	0.28–0.37	0.0	0.4771
Bone pain	4	0.33	0.24–0.43	76.1	0.0057
Arthralgia	4	0.22	0.19–0.28	9.9	0.3435
Back pain	4	0.20	0.11–0.33	85.1	0.0002
Myalgia	4	0.16	0.11–0.34	47.4	0.1272
Asthenia	4	0.12	0.09–0.16	0.0	0.8251
Musculoskeletal pain	4	0.11	0.08–0.17	53.8	0.0898
Pyrexia	3	0.14	0.07–0.25	76.7	0.0137
Pain in extremity	3	0.13	0.07–0.21	65.0	0.0575
Nausea	3	0.10	0.06–0.17	54.2	0.1127
Injection site AE	4	0.08	0.05–0.13	56.4	0.0758
Grade ≥ 3 AE	4	0.21	0.09–0.41	92.9	< 0.0001

*AE, *adverse event;* RBC, *red blood cell;* CI, *confidence interval

### Risk of bias assessment

Among the three RCTs and one single-arm trial assessed using the Cochrane ROB 2 tool, three (75%) were judged to have a low overall risk of bias, while one study (25%) raised some concerns primarily related to insufficient information about the randomization process. All studies were double-blinded, and domains related to missing outcome data, measurement of outcomes, and selective reporting were rated as low risk.

## Discussion

This meta-analysis pools current evidence on the efficacy and safety of luspatercept in beta-thalassemia from three RCTs and one single-arm trial. Luspatercept demonstrated clear clinical benefit in reducing transfusion burden, with a substantial proportion of patients achieving complete transfusion independence. The safety profile was acceptable, with most adverse effects being mild to moderate, manageable with standard care, and rarely necessitating treatment discontinuation.

### Efficacy findings

The marked reduction in RBC transfusion burden observed with luspatercept affirms its therapeutic utility beyond palliative transfusion therapy. This effect is attributable to luspatercept’s capacity to enhance late-stage erythroid maturation through modulation of transforming growth factor-beta superfamily signaling, thereby enhancing endogenous RBC production [10, 11]. The BELIEVE trial previously showed that 21.4% of luspatercept-treated patients experienced a ≥ 33% decrease in transfusion burden compared to 4.5% of placebo-treated patients, underscoring the robustness of this efficacy [9]. In transfusion-dependent β-thalassemia (TDT) patients, luspatercept elevates erythropoietic markers, lowers hepcidin, minimizes transfusion burden, and redistributes iron from macrophages to hepatocytes [12]. These reductions in transfusion requirements equate to tangible clinical benefits: decreased iron overload, diminished chelation burden, and enhanced patient quality of life.

While a minority of patients achieved complete transfusion independence, the presence of this outcome is clinically important. For patients with lifelong transfusion dependency, achieving long-term independence represents a paradigm shift in disease management. The variability in this endpoint may relate to differences in baseline bone marrow reserve, genetic modifiers, disease severity, and treatment duration.

### Safety profile

Luspatercept demonstrated a reproducible and manageable safety profile. Headache (32%) was the most commonly reported adverse event, typically mild and transient, occurring early in treatment and rarely requiring intervention. Bone pain (33%) and arthralgia (22%) were also frequently reported, possibly reflecting increased bone marrow activity with augmented erythropoiesis. Back pain (20%) was experienced and generally responded to conservative treatment. Asthenia (12%) was observed, though distinguishing treatment-induced fatigue from baseline beta-thalassemia symptoms is challenging. Injection site reactions, nausea, pain in extremities, and generalized musculoskeletal pain were generally mild and self-resolving. Grade ≥ 3 adverse events occurred in 21% of patients, underscoring the importance of patient monitoring, though severe toxicity is generally low in incidence and does not outweigh the clinical benefits for most patients [13].

### Implications for clinical practice

For clinicians managing patients with transfusion-dependent β-thalassemia, luspatercept represents an important treatment advance. The decision to initiate luspatercept should consider patient preferences regarding transfusion burden, the presence of iron overload complications, and the manageable side effect profile. Headache and bone pain are common but typically mild and transient, rarely requiring treatment discontinuation.

### Heterogeneity

The heterogeneity observed in several outcomes likely results from variation in study design, patient populations (TDT vs. NTDT), baseline disease severity, and definitions of outcomes. Despite this heterogeneity, the consistent observation of benefits across different settings amplifies the generalizability of luspatercept’s therapeutic effect.

### Limitations

This review has several limitations. First, the small number of eligible studies (*n* = 4) restricts statistical power and the precision of pooled estimates, particularly for outcomes with wide confidence intervals (e.g., transfusion independence: 95% CI 0.13–0.69). Second, heterogeneity across trials due to variance in patient populations, disease severity, study endpoints, and follow-up duration limits definitive comparative conclusions. Third, an important limitation is the lack of genotype-specific analysis. The included studies did not consistently report treatment response stratified by beta-globin genotype (e.g., β⁰/β⁰ versus β⁺/β⁺ versus β⁰/β⁺), and the small number of studies precluded meaningful subgroup analysis. Whether certain genetic subtypes predict superior response to luspatercept remains an unanswered question. Fourth, some outcomes were not consistently reported across studies. Fifth, the majority of study populations were adults, with very few data in pediatric patients. Sixth, publication bias may exist, as positive studies are more likely to be published, potentially exaggerating efficacy and underestimating harm.

## Conclusion

Luspatercept is a valuable therapeutic agent for β-thalassemia that profoundly decreases transfusion burden and, in a substantial proportion of patients, achieves complete transfusion independence. The therapy is well tolerated, with most adverse events being mild, transient, and manageable in clinical practice. Although more serious adverse effects occur infrequently, they highlight the importance of appropriate patient selection and continued monitoring. Future studies should aim to establish predictors of robust response (including genotype stratification), assess long-term outcomes, and broaden the evidence base in pediatric and non-transfusion-dependent patients. Future trials should prospectively collect and report genotype data to enable identification of genetic predictors of response, particularly for outcomes such as transfusion independence.

**Clinical Take-Home Messages**

**Table:** 

Efficacy	• 58% of patients achieve ≥ 33% reduction in transfusions • 40% achieve ≥ 50% reduction • 36% achieve transfusion independence
Safety	• Most common AEs: headache (32%), bone pain (33%), arthralgia (22%) • Grade ≥ 3 AEs occur in 21% of patients • Most AEs are mild, transient, and manageable
Clinical Implication	• Luspatercept offers a meaningful alternative to chronic transfusion therapy • Consider in patients with transfusion burden, iron overload complications, or preference to reduce transfusions

## Supplementary Information

Below is the link to the electronic supplementary material.

Supplementary Material 1

## Data Availability

No datasets were generated or analysed during the current study.
