# Supplementary material for: Outcomes with luspatercept in patients with β-thalassemia: a systematic review and meta-analysis
Source: Ann Hematol. 2026 Jun 25;105(10):411. doi: 10.1007/s00277-026-07151-5 (PMC13558420; doi:10.1007/s00277-026-07151-5)
Supplement: Supplementary file 1 [file 277_2026_7151_MOESM1_ESM.docx]

**Supplementary tables**

**Supplementary Table 1: Search strategy for each database (up to May 2025)**

| **Database** | **Search Query** | **Results** |
| --- | --- | --- |
| **PubMed** | ("luspatercept"[Title/Abstract] OR "luspatercept-aamt"[Title/Abstract] OR "RAP-536"[Title/Abstract] OR "ACE-536"[Title/Abstract] OR "reblozyl"[Title/Abstract] OR "TGF-beta superfamily ligand trap"[Title/Abstract] OR "transforming growth factor-beta superfamily ligand trap"[Title/Abstract] OR "erythroid maturation agent"[Title/Abstract]) AND ("thalassemia"[Title/Abstract] OR "thalassaemia"[Title/Abstract] OR "β-thalassemia"[Title/Abstract] OR "beta thalassemia"[Title/Abstract] OR "cooley's anemia"[Title/Abstract] OR "mediterranean anemia"[Title/Abstract] OR "β-thalassemia major"[Title/Abstract] OR "β-thalassemia minor"[Title/Abstract] OR "β-thalassemia intermedia"[Title/Abstract] OR "β-thalassemia trait"[Title/Abstract] OR "beta-thalassemia major"[Title/Abstract] OR "beta-thalassemia minor"[Title/Abstract] OR "beta-thalassemia intermedia"[Title/Abstract] OR "transfusion-dependent thalassemia"[Title/Abstract] OR "TDT"[Title/Abstract] OR "non-transfusion-dependent thalassemia"[Title/Abstract] OR "NTDT"[Title/Abstract] OR "hydrops fetalis"[Title/Abstract] OR "hemoglobin H disease"[Title/Abstract] OR "HbH disease"[Title/Abstract]) AND (randomized controlled trial[Publication Type] OR controlled clinical trial[Publication Type] OR clinical trial[Publication Type]) | 86 |
| **Scopus** | ( TITLE-ABS-KEY("luspatercept" OR "luspatercept-aamt" OR "RAP-536" OR "ACE-536" OR "reblozyl" OR "TGF-beta superfamily ligand trap" OR "transforming growth factor-beta superfamily ligand trap" OR "erythroid maturation agent") ) AND ( TITLE-ABS-KEY("thalassemia" OR "thalassaemia" OR "β-thalassemia" OR "beta thalassemia" OR "cooley's anemia" OR "mediterranean anemia" OR "β-thalassemia major" OR "β-thalassemia minor" OR "β-thalassemia intermedia" OR "β-thalassemia trait" OR "beta-thalassemia major" OR "beta-thalassemia minor" OR "beta-thalassemia intermedia" OR "transfusion-dependent thalassemia" OR "TDT" OR "non-transfusion-dependent thalassemia" OR "NTDT" OR "hydrops fetalis" OR "hemoglobin H disease" OR "HbH disease") ) AND ( LIMIT-TO(DOCTYPE , "ar") ) AND ( LIMIT-TO(LANGUAGE , "English") ) | 170 |
| **Embase** | ('luspatercept'/exp OR 'luspatercept' OR 'luspatercept-aamt' OR 'rap-536' OR 'ace-536' OR 'reblozyl' OR 'tgf beta superfamily ligand trap' OR 'erythroid maturation agent') AND ('thalassemia'/exp OR 'thalassemia' OR 'thalassaemia' OR 'beta thalassemia' OR 'cooley anemia' OR 'mediterranean anemia' OR 'transfusion dependent thalassemia' OR 'tdt' OR 'non transfusion dependent thalassemia' OR 'ntdt') AND ([randomized controlled trial]/lim OR [controlled clinical trial]/lim OR [clinical trial]/lim) AND [english]/lim | 248 |
| **Cochrane Library** | ("luspatercept" OR "luspatercept-aamt" OR "RAP-536" OR "ACE-536" OR "reblozyl" OR "TGF-beta superfamily ligand trap" OR "transforming growth factor-beta superfamily ligand trap" OR "erythroid maturation agent") AND ("thalassemia" OR "thalassaemia" OR "β-thalassemia" OR "beta thalassemia" OR "cooley's anemia" OR "mediterranean anemia" OR "transfusion-dependent thalassemia" OR "TDT" OR "non-transfusion-dependent thalassemia" OR "NTDT") in Title Abstract Keyword | 65 |
| [**ClinicalTrials.gov**](https://clinicaltrials.gov/) | (luspatercept OR luspatercept-aamt OR RAP-536 OR ACE-536 OR reblozyl) AND (thalassemia OR beta thalassemia OR TDT OR NTDT) | 18 |
| **Total** |  | **587** |

**Supplementary Figures**

**Supplementary Figure S1: Forest plot of arthralgia prevalence**

Forest plot showing the pooled proportion of patients reporting arthralgia as an adverse event with luspatercept. Four studies contributed to this analysis (Piga 2019, Piga 2022, Cappellini 2022, Cappellini 2025). Pooled proportion: 0.22 (95% CI: 0.19–0.28). Heterogeneity: I² = 9.9%, p = 0.3435, indicating low heterogeneity across studies.

**Supplementary Figure S2: Forest plot of asthenia prevalence**

Forest plot showing the pooled proportion of patients reporting asthenia (fatigue or lowered energy levels) as an adverse event with luspatercept. Four studies contributed to this analysis (Piga 2019, Piga 2022, Cappellini 2022, Cappellini 2025). Pooled proportion: 0.12 (95% CI: 0.09–0.16). Heterogeneity: I² = 0.0%, p = 0.8251, indicating no heterogeneity across studies.

**Supplementary Figure S3: Forest plot of back pain prevalence**

Forest plot showing the pooled proportion of patients reporting back pain as an adverse event with luspatercept. Four studies contributed to this analysis (Piga 2019, Piga 2022, Cappellini 2022, Cappellini 2025). Pooled proportion: 0.20 (95% CI: 0.11–0.33). Heterogeneity: I² = 85.1%, p = 0.0002, indicating high heterogeneity across studies.

**Supplementary Figure S4: Forest plot of bone pain prevalence**

Forest plot showing the pooled proportion of patients reporting bone pain as an adverse event with luspatercept. Four studies contributed to this analysis (Piga 2019, Piga 2022, Cappellini 2022, Cappellini 2025). Pooled proportion: 0.33 (95% CI: 0.24–0.43). Heterogeneity: I² = 76.1%, p = 0.0057, indicating high heterogeneity across studies.

**Supplementary Figure S5: Forest plot of myalgia prevalence**

Forest plot showing the pooled proportion of patients reporting myalgia (muscle pain) as an adverse event with luspatercept. Four studies contributed to this analysis (Piga 2019, Piga 2022, Cappellini 2022, Cappellini 2025). Pooled proportion: 0.16 (95% CI: 0.11–0.34). Heterogeneity: I² = 47.4%, p = 0.1272, indicating moderate heterogeneity across studies.

**Supplementary Figure S6: Forest plot of musculoskeletal pain prevalence**

Forest plot showing the pooled proportion of patients reporting musculoskeletal pain as an adverse event with luspatercept. Four studies contributed to this analysis (Piga 2019, Piga 2022, Cappellini 2022, Cappellini 2025). Pooled proportion: 0.11 (95% CI: 0.08–0.17). Heterogeneity: I² = 53.8%, p = 0.0898, indicating high heterogeneity across studies.

**Supplementary Figure S7: Forest plot of injection site adverse effects**

Forest plot showing the pooled proportion of patients reporting injection site adverse effects (e.g., pain, redness, swelling) with luspatercept. Four studies contributed to this analysis (Piga 2019, Piga 2022, Cappellini 2022, Cappellini 2025). Pooled proportion: 0.08 (95% CI: 0.05–0.13). Heterogeneity: I² = 56.4%, p = 0.0758, indicating high heterogeneity across studies.

**Supplementary Figure S8: Forest plot of nausea prevalence**

Forest plot showing the pooled proportion of patients reporting nausea as an adverse event with luspatercept. Three studies contributed to this analysis (Piga 2019, Cappellini 2022, Cappellini 2025). Pooled proportion: 0.10 (95% CI: 0.06–0.17). Heterogeneity: I² = 54.2%, p = 0.1127, indicating high heterogeneity across studies.

**Supplementary Figure S9: Forest plot of pain in extremity prevalence**

Forest plot showing the pooled proportion of patients reporting pain in the extremity (arm or leg pain) as an adverse event with luspatercept. Three studies contributed to this analysis (Piga 2019, Cappellini 2022, Cappellini 2025). Pooled proportion: 0.13 (95% CI: 0.07–0.21). Heterogeneity: I² = 65.0%, p = 0.0575, indicating high heterogeneity across studies.

**Supplementary Figure S10: Forest plot of pyrexia prevalence**

Forest plot showing the pooled proportion of patients reporting pyrexia (fever) as an adverse event with luspatercept. Three studies contributed to this analysis (Piga 2019, Cappellini 2022, Cappellini 2025). Pooled proportion: 0.14 (95% CI: 0.07–0.25). Heterogeneity: I² = 76.7%, p = 0.0137, indicating high heterogeneity across studies.

**Supplementary Figure S11: Forest plot of hemoglobin increase ≥1.0 g/dL**

Forest plot showing the pooled proportion of patients achieving a mean increase in hemoglobin of ≥1.0 g/dL with luspatercept. Three studies contributed to this analysis (Piga 2019, Piga 2022, Cappellini 2022). Pooled proportion: 0.69 (95% CI: 0.53–0.81). Heterogeneity statistics were not reported for this outcome in the source data.
